# The moving minimum audible angle is smaller during self motion than during source motion

**DOI:** 10.3389/fnins.2014.00273

**Published:** 2014-09-02

**Authors:** W. Owen Brimijoin, Michael A. Akeroyd

**Affiliations:** Scottish Section, Institute of Hearing Research, Medical Research Council/Chief Scientist OfficeGlasgow, UK

**Keywords:** spatial hearing, head movements, auditory motion, sound localization, motion tracking, self-motion compensation

## Abstract

We are rarely perfectly still: our heads rotate in three axes and move in three dimensions, constantly varying the spectral and binaural cues at the ear drums. In spite of this motion, static sound sources in the world are typically perceived as stable objects. This argues that the auditory system—in a manner not unlike the vestibulo-ocular reflex—works to compensate for self motion and stabilize our sensory representation of the world. We tested a prediction arising from this postulate: that self motion should be processed more accurately than source motion. We used an infrared motion tracking system to measure head angle, and real-time interpolation of head related impulse responses to create “head-stabilized” signals that appeared to remain fixed in space as the head turned. After being presented with pairs of simultaneous signals consisting of a man and a woman speaking a snippet of speech, normal and hearing impaired listeners were asked to report whether the female voice was to the left or the right of the male voice. In this way we measured the moving minimum audible angle (MMAA). This measurement was made while listeners were asked to turn their heads back and forth between ± 15° and the signals were stabilized in space. After this “self-motion” condition we measured MMAA in a second “source-motion” condition when listeners remained still and the virtual locations of the signals were moved using the trajectories from the first condition. For both normal and hearing impaired listeners, we found that the MMAA for signals moving relative to the head was ~1–2° smaller when the movement was the result of self motion than when it was the result of source motion, even though the motion with respect to the head was identical. These results as well as the results of past experiments suggest that spatial processing involves an ongoing and highly accurate comparison of spatial acoustic cues with self-motion cues.

## Introduction

Listeners make continual head movements, be they intentional head turns, reflexive orienting responses, or small involuntary movements. Because the ears are attached to the head and the head is never perfectly still, this means that the acoustic world must also be in constant motion. We nonetheless perceive the auditory world to be relatively stable. The underlying mechanisms that permit this percept are unknown. The visual system incorporates a low-level mechanism that counteracts the motion of the head, the “vestibulo-ocular reflex” (VOR). Using input from the vestibular and proprioceptive systems, the VOR works to physically move the eyes in direct opposition to one's own head motion, more or less stabilizing the projection of images on the surface of the retina (Lorente De No, 1933). Such a mechanical solution is not possible in the auditory system due to the simple fact that the ears are fixed to the sides of the head. Thus each time the head turns, the acoustic world at the ears turns in the opposite direction. We refer to this as “self motion” and contrast it with “source motion”: that which is due to the source of sound itself moving.

While both head motions and physically moving sound sources in the world result in acoustic movement at the ears, self motion is not perceived as a moving sound: simple introspection will demonstrate that the acoustic world appears to remain relatively stable as the head turns. By analogy with the VOR, it is sensible to suggest that there exists a fundamental mechanism by which the moving auditory world is perceptually stabilized (Lewald and Karnath, 2000; Lewald et al., 2000). Evidence directly supporting such a mechanism, however, remains somewhat circumstantial, despite there being a wealth of studies showing a tight integration between motion and auditory spatial perception in general. Heads are essentially in continual motion (König and Sussman, 1955) and movements have been shown to increase the accuracy of sound localization judgments (Thurlow and Runge, 1967; Perrett and Noble, 1997); in particular they have been shown to play a critical role in resolving the front/back position of a sound source (Wightman and Kistler, 1999; Brimijoin and Akeroyd, 2012; Kim et al., 2013). Head motions have also been linked to the degree to which sound sources are externalized (Brimijoin et al., 2013). Vestibular stimulation has been shown to shift a listener's subjective auditory midline (Lewald and Karnath, 2000); and, in a complementary fashion, rotating auditory stimuli can induce an illusion of rotational self motion (Lackner, 1977). A number of related studies are discussed at the end of this manuscript, but here it should be noted that together they suggest that vestibular information is thoroughly integrated with auditory spatial information. To our knowledge, however, no study has directly tested whether self motion is processed differently from source motion, nor has any examined the impact of compensation for self-generated motion.

If there is an auditory stabilization mechanism that works to at least partially cancel out self-generated movements, it is reasonable to expect that it would provide a more stable background against which a listener could judge the position and/or motion of auditory sources. Such a scenario leads to the following prediction: listeners' performance on moving spatial auditory tasks should be better when the acoustic movement in question is generated by their own motion than when it is generated by the source itself. We tested this prediction using a measurement of the *moving* minimum audible angle (MMAA), which we define to be the smallest angular separation between two simultaneous, moving sound sources that is needed for a listener to be able to tell that the two sources are in separate directions. The MMAA is a generalization of the classical minimum audible angle (MAA), which uses sounds that are static (Mills, 1958). Also, the MMAA should not be confused with the minimum audible movement angle (MAMA), which is the smallest detectable motion of a sound (Perrott and Tucker, 1988). We measured the MMAA using two simultaneous signals, separated in space rather than sequential signals, marking a slight departure from traditional methods of measuring the MAA (though note that the MAA for concurrent sounds has been measured previously; Perrott, 1984).

The use of high speed infrared motion tracking (see Brimijoin and Akeroyd, 2012) allowed us to tightly control the movement of virtual signals. In this way we were able to measure the performance of listeners when presented with self motion vs. source motion while ensuring that the actual movement itself was identical in the two conditions. We found an advantage for spatial processing during movement when the movement in question was self motion rather than source motion. This advantage was similar in size across a wide range of ages and levels of hearing impairment.

## Methods

### Listeners

**Figure 1:**
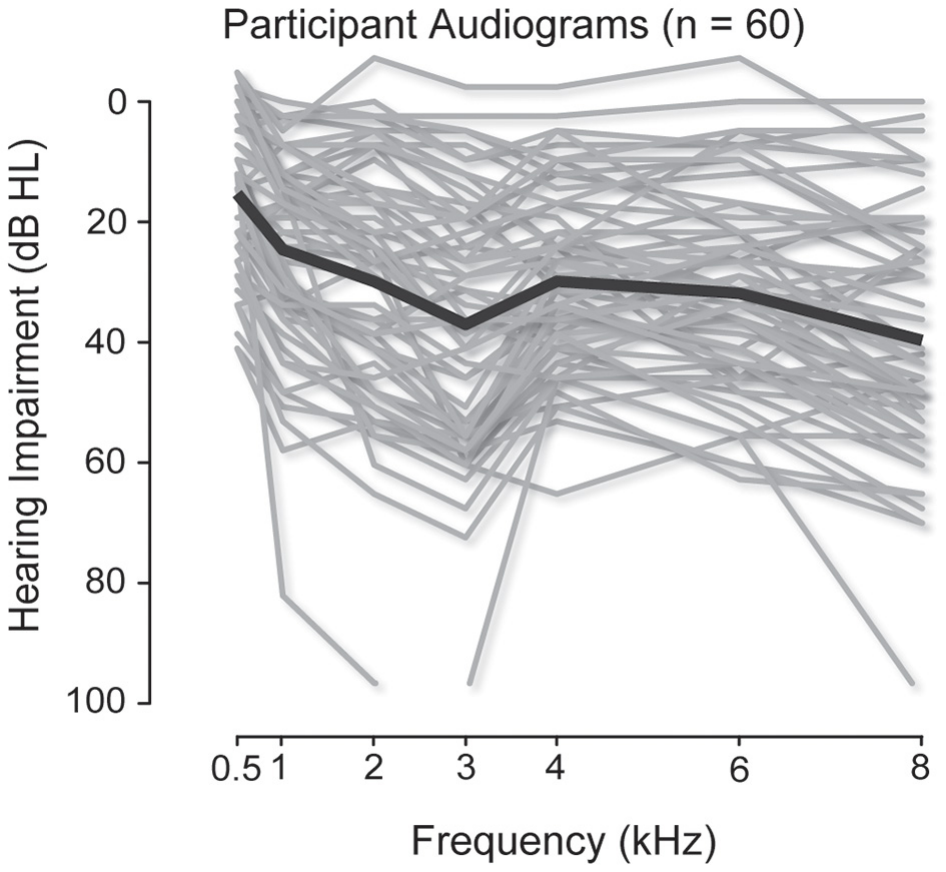
**Audiograms for the participants**. The individual audiograms, measured at 500, 1000, 2000, 3000, 4000, 6000, and 8000 Hz, of all listeners are plotted in gray. The mean audiograms are plotted as solid black lines.

We recorded complete data sets (i.e., had successful motion tracking throughout all conditions) and made MAA and MMAA measurements for 60 listeners. Audiograms for the complete subject pool are shown in Figure 1. The individual audiogram in decibels Hearing Level (dB HL) of each listener is plotted in gray and the mean for all listeners is plotted as a solid black line. Hearing thresholds were measured at 250, 500, 1000, 2000, 3000, 4000, 6000, and 8000 Hz for both left and right ears. For the purposes of analysis, mean thresholds were computed for each ear by averaging the hearing threshold at 500, 1000, 2000, and 4000 Hz. All listeners had less than 15 dB of difference in their mean hearing thresholds between the two ears.

Each listener was seated in a quiet, sound-treated room and presented with pairs of simultaneous signals over headphones consisting of a man and a woman speaking 1-s duration snippets of speech. The sentence fragments were drawn from the Adaptive Sentence List corpus (Macleod and Summerfield, 1987); these sentences were sampled at 44.1 kHz, but low pass filtered at 10 kHz, and presented at a comfortable listening level (typically between 65 and 80 dB sound pressure level). The signals were processed using virtual acoustics to appear to come from different directions. For each of the four conditions (see below) the male and female voices differed in direction by any of 10 values (±1°, 2°, 4°, 8°, and 16°, chosen pseudo-randomly on each trial) and the mean presentation angle of the two signals was randomly varied across five angles (−16°, −8°, 0°, 8°, and 16°, where 0° corresponds to directly in front, negative to the left, and positive to the right). The listeners were asked to determine whether the female voice was to the left or the right of the male voice, regardless of the pair's absolute position in space. The order of male-female separation angle within the conditions was randomized, but within blocks listeners always performed the head-moving condition first, as the trajectories measured here were used in the source-motion condition.

### Motion tracking

Motion tracking was performed in a sound-treated room using a commercial infrared camera system (Vicon MX3+) using methods described previously (Brimijoin et al., 2010). Six cameras were placed above the listener, behind and ahead, and were pointed toward the listener. The system tracked 9-mm diameter reflective spheres; these “markers” were placed on a head-mounted “crown” worn by the listeners. The motion-tracking system was queried from Matlab and returned three-dimensional Cartesian coordinates of the crown markers at a sample rate of 100 Hz. Arctangent transforms converted these coordinates to the three Euler angles of yaw, pitch, and roll. These angles were accurate to within approximately 0.1°.

### Virtual sound field reproduction

We used linearly-interpolated manikin (KEMAR, Burkhard and Sachs, 1975) binaural room impulse responses (BRIR), measured at 1.0 m, to create virtual sound locations in the horizontal plane (Wierstorf et al., 2011). When a signal is filtered using BRIRs and played back over headphones, the result is audio that seems to be emanating from a particular direction relative to the head. The use of BRIRs instead of free-field loudspeakers allowed us to create two directly comparable experimental conditions. To measure self- vs. source-motion acuity using loudspeakers would require a comparison between statically presented signals while the head was moving, and dynamically panned signals while the head was kept still. As the signal processing in these two cases is different, we opted to use virtual acoustics to create acoustically identical conditions that differed only in whether the presented motion was specified relative to the head or relative to the world. It should be noted that the use of generic BRIRs recorded solely in the horizontal plane does carry with it two drawbacks: (1) the realism of the simulation was partly dependent on the similarity of the participant's head to that of the KEMAR manikin; and (2) neither head rotations in the vertical plane nor head translations were accounted for, potentially decreasing the realism of the acoustic simulation, and/or the perceived source elevation (although listeners were given feedback on the ideal head movements during a trial period). The use of this database, however, allowed us to create two experimental conditions that were acoustically identical to one another without the complexity and time requirements associated with measuring hundreds of individualized BRIRs.

Every 10 ms, the listener's head direction was measured by the motion tracking system and the two closest BRIRs from the database were selected and then linearly interpolated with one another to give a BRIR corresponding to the actual direction. The interpolation was performed as a weighted sum in the time domain. This technique was computationally efficient enough to allow us to do real time processing, but could in principle result in interpolated BRIRs with doubled attenuated peaks. We largely avoided this problem by using a BRIR library that was measured in 1° intervals (Wierstorf et al., 2011), meaning that the time difference between angle-adjacent BRIRs was smaller than the sample period (1/44100)^1^. The interpolated 512-sample long BRIR was then convolved with a 512-sample long chunk of audio and the last 441 samples (corresponding to 10 ms) were sent to an audio buffer. The time position in the acoustic signal was then incremented by 441 samples and the process was repeated. Transitions between buffer segments were smoothed using a 32-sample linear crossfade. The audio buffering was handled using playrec (www.playrec.co.uk), a custom Matlab audio protocol built on the PortAudio API. All together, these methods could change the virtual location of two audio signals every 10 ms with a total movement-to-change latency of between 22 and 33 ms. Our experience was that the method was smooth, and none of the sounds had perceptible jumps, transitions, or clicks.

### Statistical analysis

The data across the 10 values of male-female separation angles for each condition defined a psychometric function for percent-correct vs. separation angle. The absolute values of the separation of the male and female voice (i.e., positive vs. negative subtended angles) were averaged to yield five points on each psychometric function. These were fitted with a logistic function using “nlinfit” from the Statistics Toolbox for Matlab release 2012a (The Mathworks, Natick MA). Values of MMAA, defined as the separation angle needed to give a performance of 75%, were calculated from the logistic fits. We used SPSS v21 (IBM, Armonk NY) to perform an ANOVA on the MMAA values as a function of listening condition. We made two *post-hoc* comparisons to determine whether there were significant differences between the two static-signal conditions and between the two moving-signal conditions. Alpha was set to 0.05 for the ANOVA and the Bonferroni correction was used for all *post-hoc* tests.

### Procedure

**Figure 2:**
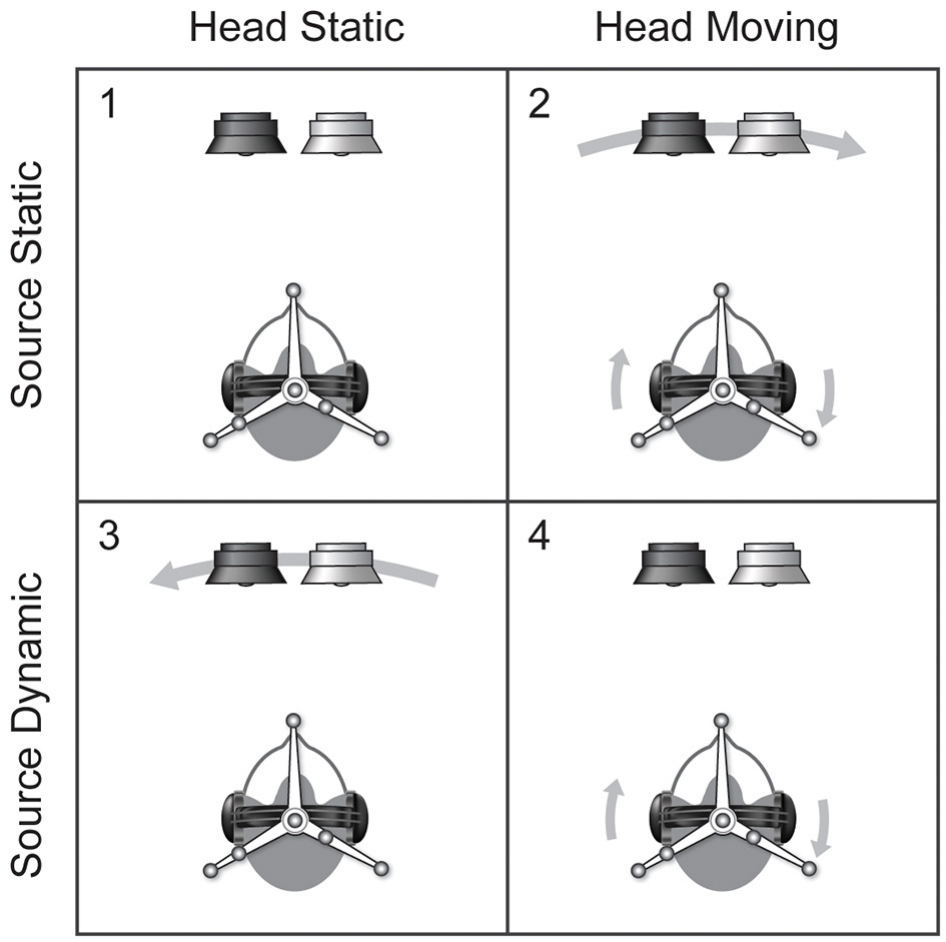
**The 2 × 2 experiment design**. Tests of the minimum audible angle were conducted in four conditions: **(1)** listeners were asked to remain static and the signal was not moved, **(2)** listeners were asked to turn their heads and the signal moved with them, **(3)** listeners were asked to remain static and the signals were moved, and **(4)** listeners were asked to turn their heads and the signal was adjusted so as to appear to remain still with respect to the world.

We ran four sets of conditions (Figure 2). In all conditions the listeners were asked to report the relative position of the female voice with respect to the male voice. In two of the conditions, we asked listeners to remain still in the ring of loudspeakers, in the other two, the listener was asked to turn his/her head back and forth continually between two visual markers at ±15° while we used motion tracking to determine the orientation of the listener's head every 10 ms. The listeners were given feedback until their rotations were within a few degrees of this target motion and their peak velocities were roughly 45°/s.

In one of the four conditions, the self-motion condition, the pair of signals were dynamically filtered so that they appeared to remain fixed at particular absolute directions with respect to the world as the listeners turned (see Brimijoin et al., 2013 for a more complete methods description). In the source-movement condition, listeners were asked to remain still and were played signals that moved according to randomly chosen motion trajectories recorded in the self-motion condition. It should be noted that although listeners were asked to remain still, our experience is that they still made continual micromotions. These <0.5° head motions aside, the signals in the self-motion and source-motion conditions shared the identical acoustic movements, but the movement was in the first instance perfectly correlated with the listener's own head motion (self motion) whereas in the second it was entirely uncorrelated (source motion). We also ran two control conditions, in which the signals did not use dynamically interpolated BRIRs but instead were fixed with respect to the head, whether the listener was static or moving. Note that only in the head static/signal static condition does our measurement correspond to a classic simultaneous MAA.

Thus the experiment was conducted using a 2 × 2 design in which listeners were asked to either remain static or to move their heads and presented with pairs of signals that were either static or moving with respect to the head (see Figure 2). The four conditions were: (1) head static/source static, (2) head moving/source static, (both 1 and 2 being standard headphone presentation), (3) head static/source dynamic (source motion), and (4) head moving/source dynamic (self motion).

## Results

### Psychometric functions

**Figure 3:**
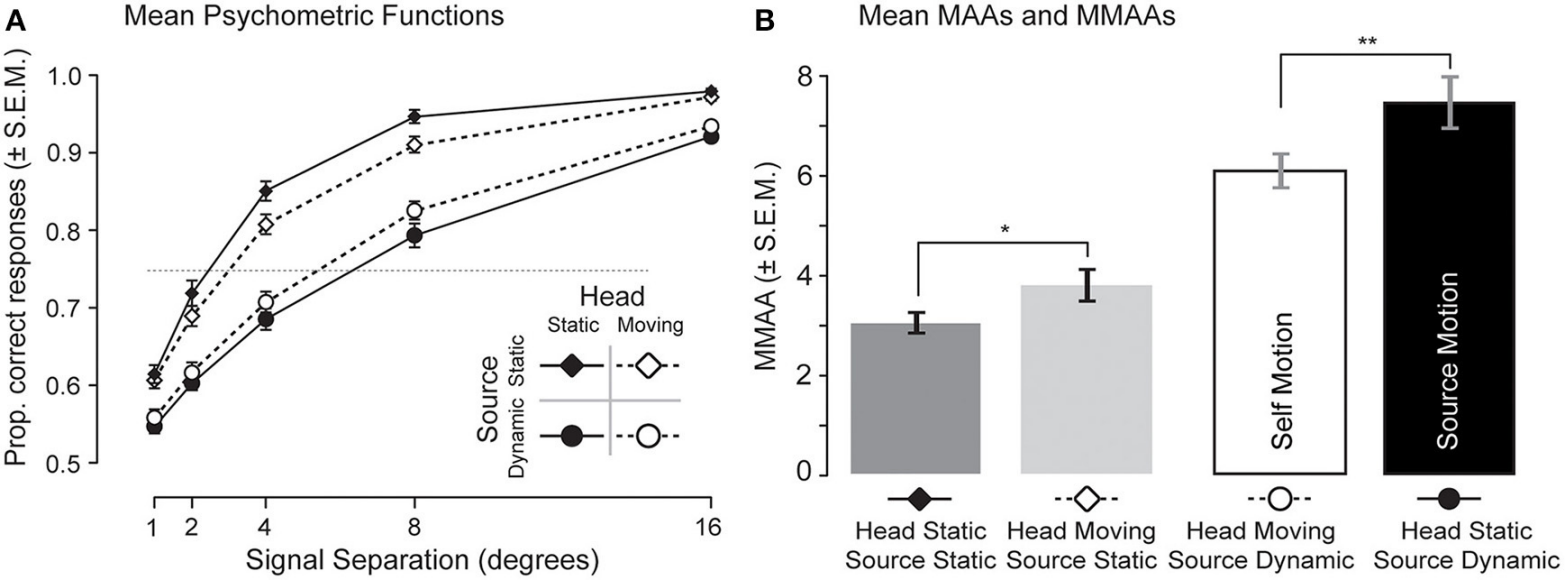
**Psychometric functions and measurements of the minimum audible angle. (A)** The mean psychometric functions for all listeners are plotted for all four conditions including: (1) head static/source static (solid diamonds), (2) head moving/source static (open diamonds), (3) head static/source dynamic (solid circles), and (4) head moving/source dynamic (open circles). Error bars show ±1 standard error. **(B)** The results of logistic fits to individual psychometric functions show that the MMAA was larger for source motion (black bar) than for self motion (white bar), and that both of these were larger than for the source static conditions (gray bars). A single asterisk represents differences in means significant at an alpha of 0.05, and a double asterisk an alpha of 0.01.

The across-listener mean psychometric functions of proportion correct relative localization are shown in Figure 3A. For all conditions, the mean proportion correct increased as a function of the separation of the male and female voices. While the 75% threshold differences are reported below, it may be observed that the difference in performance between the two signal static conditions (top two curves) suggests that listeners were most easily able to discriminate the left/right positions of separated signals when both the listener and the signals did not move (solid diamonds). When the listeners were required to turn their heads back and forth and the signal moved with them (open diamonds), their mean performance appeared to drop. The offset in the two “source dynamic” curves suggests that listeners were better able to discriminate the position of signals that moved in realistic opposition to their head movements (open circles) than those that appeared to move arbitrarily in space (solid circles).

### Minimum audible angle measurements

Figure 3B plots the mean MMAAs calculated from the logistic fits to each listener's psychometric functions. A Two-Way repeated measures ANOVA confirmed a significant main effect of signal type (static vs. dynamic) [*F*_(3,236)_ = 195.7, *p* < 0.001]. This result is due to the large difference between the MAA measurements and the MMAA measurements, arguing that the dynamically moving signals were associated with an increased difficulty in discriminating the two target signal positions. The ANOVA revealed no effect of head movement [*F*_(3,236)_ = 4.4, *p* = 0.40] but a significant interaction between movement and signal type [*F*_(1,236)_ = 52.9, *p* < 0.005]. The lack of a main effect of head movement is due to an opposite influence of head movement seen in the two signal conditions.

In the “signal static” conditions, the MAAs were between 3 and 4°, but in the self-motion condition they averaged 5.4°, and in the source-motion condition they averaged 6.6°. *Post-hoc* contrasts using a Bonferonni correction revealed a significant difference in MMAAs between the two dynamic signal conditions (a mean difference of 1.2°, *p* < 0.01). A *post-hoc* test also showed that there was a significant difference between the two signal-static conditions (mean difference in MMAA of 0.7°, *p* < 0.05), arguing that signals fixed relative to the head were less easily discriminated in position when the listener was moving than they were when the listener was static.

**Figure 4:**
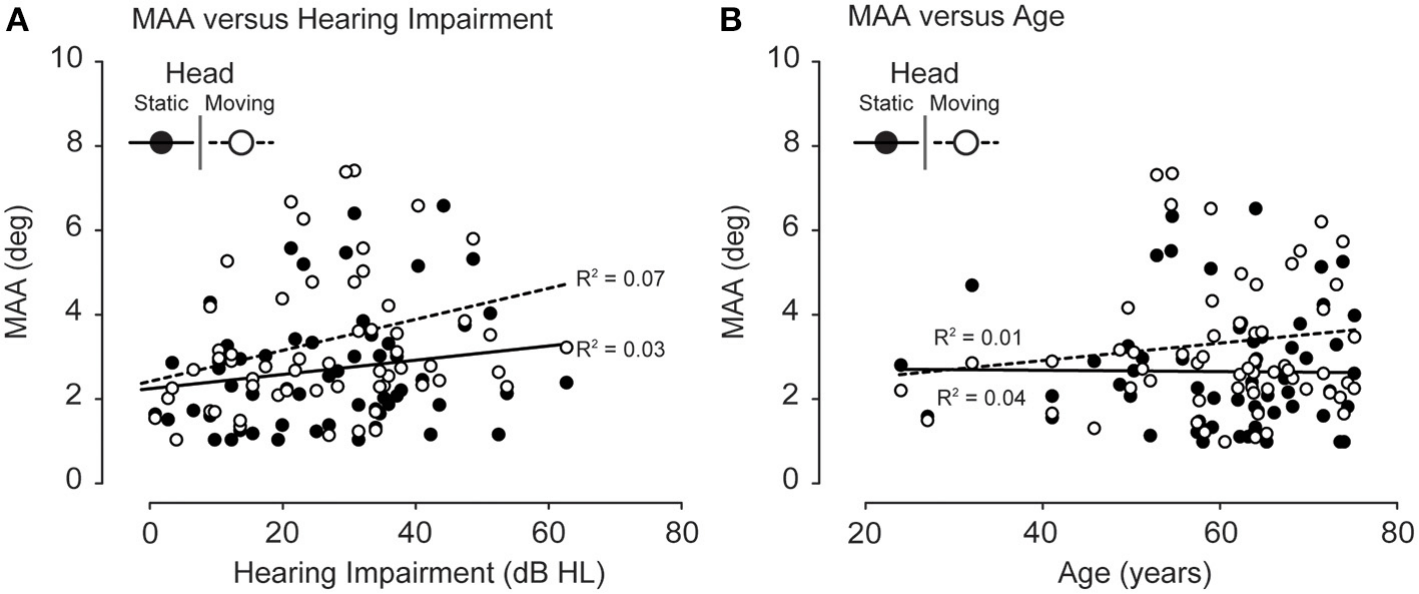
**(A)** Static MAA as a function of hearing impairment. No increase in MAA was observed as a function of hearing impairment for either the head moving (open circles, dotted line) or the head static conditions (filled circles, solid line). **(B)** MAA as a function of age. No increase in MAA was observed as a function of age for either the head moving (open circles, dotted line) or the head static conditions (filled circles, solid line).

The classical, static-signal MAA has been previously shown to increase as a function of hearing impairment (Häusler et al., 1983). Figure 4A shows the results of an attempt to replicate this finding, plotting MAA for static signals as a function of hearing impairment. While the variance in MAA measurements appears to increase as a function of hearing impairment, an *R*^2^ value of 0.03 for static heads and an *R*^2^ of 0.07 for moving heads suggests that the mean MAA for statically presented signals did not increase with level of hearing impairment. The discrepancy between these results and those of Häusler et al. (1983) may be due to the fact that our measurements of the MAA were all made in front of the listener, rather than off to the sides where Häusler et al. observed the greatest effect of hearing impairment. Figure 4B plots the mean MAA values as a function of age and also showed no significant correlations (*R*^2^ of 0.04 and 0.01 for head static and head moving, respectively).

**Figure 5:**
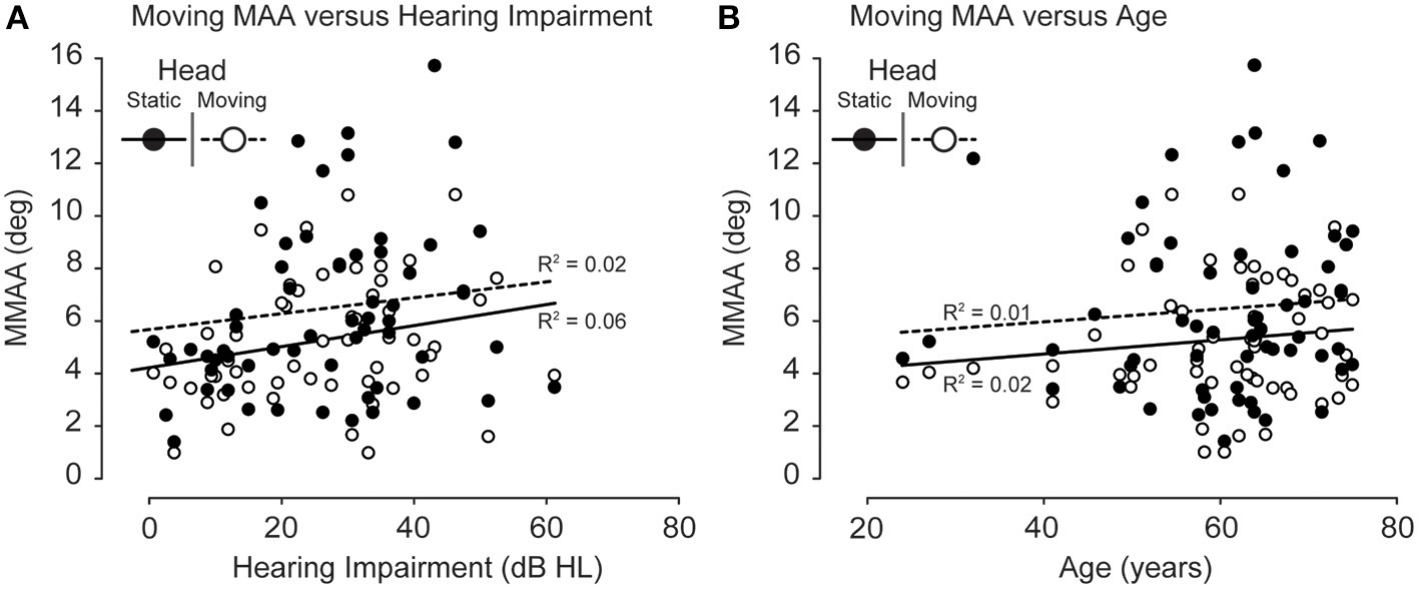
**(A)** MMAA as a function of hearing impairment. No increase in MMAA was observed as a function of hearing impairment for either the head-moving (open circles, dotted line) or head-static (filled circles, solid line) conditions. **(B)** MMAA as a function of age. No increase in MMAA was observed as a function of age for either the head-moving (open circles, dotted line) or the head-static conditions (filled circles, solid line).

For the dynamic signal stimuli (whether self- or source motion), the MMAA for dynamic sounds also did not increase with hearing impairment (Figure 5A). There was an apparent increase in variance as a function of hearing impairment, but the correlations were low for both conditions [*R*^2^ of 0.02 and 0.06 for self motion (head moving) and source motion (head static), respectively]. Apart from a similar increase in variance, no effect of age was found for the MMAA either (Figure 5B) (*R*^2^ of 0.02 and 0.01 for self motion and source motion, respectively).

### Condition-dependent differences in the minimum audible angle

**Figure 6:**
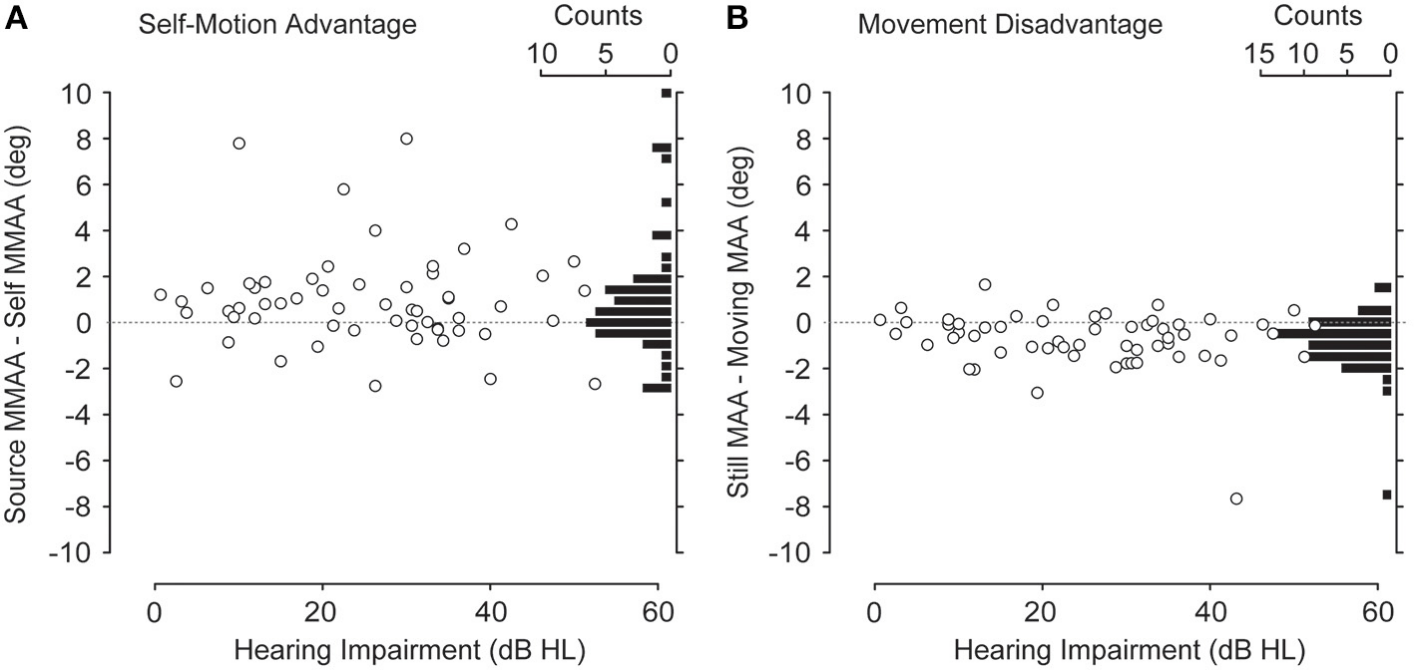
**(A)** Self-motion advantage. The open circles are the MMAAs measured during self motion subtracted from the MMAAs measured during source motion. The histograms to the right are binned data. The majority of points fall above the zero difference line (dotted line). **(B)** Movement disadvantage. The open circles are MAAs measured during head movements subtracted from the MAAs measured while listeners kept their heads still. The histograms to the right are binned data. The majority of points fall below the zero difference line (dotted line).

Figure 6A plots the difference in MMAA between the source-motion and self-motion conditions (the “self-motion advantage”) as a function of hearing impairment plotted as open circles with a histogram in 0.5° bins on the right y-axis. The majority of the points fall above the zero line (also shown by the distribution of the histogram), confirming the slight advantage for processing self motion, although there was no consistent effect of level of hearing impairment on the self-motion advantage. A similar analysis may be found in Figure 6B, but in this case these data are the difference between the MAAs found in the two signal static conditions. The consistent pattern is that there was a slight disadvantage in MAA performance when listeners were required to turn their heads and the acoustic world moved with them (a histogram of these data points is found on the right y-axis). No effect of level of hearing impairment on this difference was found.

## Discussion

Listeners had lower MMAAs when the signals moved in a way that was correlated with the listener's own movement (self motion) compared to when they were uncorrelated (source motion). These results demonstrate that there is a relative advantage in spatial processing when listeners are tested using self motion as compared to source motion. This advantage is maintained even in the older, impaired auditory system, as is evidenced by the consistent difference in the self- vs. source-motion conditions across listeners of all levels of hearing impairment and age. These data are consistent with the hypothesis that the percept of sound source location is at least partially corrected for self-generated motion, providing a more stable background against which a listener can judge the position of auditory sources.

Despite the evidence for a self-motion processing advantage, there appeared to be a consistent *disadvantage* associated with head movement for the signal static conditions (see Figure 6B). There are two possible explanations for this phenomenon, the first is that requiring listeners to move makes them less able to process spatial cues, the second is that the disadvantage is the result of the signals not moving in a behaviorally relevant manner (i.e., moving with the head). The first would imply that the advantage observed in MMAA processing is an underestimation of the true self-motion advantage, since listeners in the self-motion condition were required to turn their heads, incurring an obligatory movement penalty^2^. The second explanation is that the synchronous movement of the auditory world with the head in the signal static conditions causes a mismatch between the expected and actual movement of the signal. We have previously demonstrated that auditory externalization drops significantly when sound fields are artificially moved with the head (Brimijoin et al., 2013), a finding that was interpreted to be due to the mismatch between the movement of the head and the movement of the signal. Such a mismatch could also be responsible for the apparent MMAA movement disadvantage observed in the current study. Given the evidence of the impact of unrealistic sound field movement on auditory externalization, we feel that the second explanation for the motion disadvantage is more likely.

It should be noted that listeners were better able to determine the relative position of two sound sources when they were statically presented as compared to when they were dynamically moved. This impact on performance was consistent whether listeners moved their heads or not. The difference could be attributable to two factors: first that our method of dynamically adjusting the location of signals in virtual acoustic space resulted in a more diffuse, or “smeared” location percept, and second, that it is more difficult to judge the relative position of two simultaneously moving signals. Given our current data set, we are unable to assess the relative validity of these two explanations.

More generally speaking, however, to compare the movement of the head with the movement of the acoustic world would require both accurate auditory spatial processing and accurate processing of self motion. There is extensive interconnection between the central vestibular and auditory systems (Abraham et al., 1977), starting as low as the cochlear nucleus (Burian and Gstoettner, 1988), so making it likely that the auditory system incorporates vestibular input at multiple stages of processing. Indeed one could argue that the lack of a clearly defined vestibular cortex responding exclusively to vestibular signals (Guldin and Grüsser, 1998; Chen et al., 2010) would suggest that vestibular information is heavily integrated into the other senses prior to or in conjunction with the arrival of sensory input to the cortex. There are documented interactions between vestibular input and auditory spatial perception, such as the “audiogyral illusion” (Clark and Graybiel, 1949; Lester and Morant, 1970): when listeners are seated in a rotating room, their spatial auditory localization is shifted in the opposite direction from the rotation of the room. A related phenomenon, known as the “audiogravic illusion” (Graybiel and Niven, 1951), demonstrates that linear acceleration affects sound localization by shifting the perceived location of signals opposite to the acceleration of the listener. These studies provide evidence that physical motion can cause spatial auditory displacement. It has also been shown that moving sounds can induce the percept of self motion (for a review see Väljamäe, 2009). Taken together with the common observation that the world does not seem to spin in the opposite direction as one's head turns, the present evidence, allied to the previous data, becomes compelling: vestibular input is on some level deeply linked to auditory input.

Whether, however, the vestibular system works to simply subtract one's own motion from the movement of the acoustic world is a more difficult hypothesis to test. The eye movement driven by the vestibular-ocular reflex that largely subtracts self motion from the visual world can be easily observed, whereas any self-motion subtraction that might exist in the auditory system must be accomplished computationally, rendering it more problematic to observe experimentally. The evidence for a self-motion advantage presented in the current study is *suggestive* of a subtraction, but cannot be considered prima facie evidence for such a mechanism. Eye movements, for example, likely play a role in spatial auditory coordinate transformation. Strict geometric rules govern how the position of real world acoustic signals change with respect to the position and angle of the head (Wallach, 1940), none of which are in any way affected by the position of the eyes, but eye position has been shown to affect spatial localization (Lewald and Ehrenstein, 1996; Lewald, 1997, 1998). Furthermore, eye position influences audiovestibular interaction as well (Van Barneveld and Van Opstal, 2010), arguing that on some level that the primary driver of self-motion subtraction may be the eye movement itself. Certainly the best understood auditory spatial coordinate transformation is that which is driven by eye position. Psychophysically this transformation was described in the 1990s (Lewald and Ehrenstein, 1996; Lewald, 1997, 1998) and there is a growing body of physiological work that compliments this behavioral work. For example, the responses of neurons in the inferior colliculus have been shown to be modulated by eye position (Groh et al., 2001; Zwiers et al., 2004). Auditory receptive fields of neurons in the superior colliculus have also been shown to shift with eye position in both cats (Jay and Sparks, 1984, 1987) and primates (Hartline et al., 1995). An interaction between eye movements and head movements surely plays a role in spatial auditory processing, but we did not track the eye position of our participants. We asked listeners to fixate at a point directly ahead of their bodies as they turned their heads. Since we did not use an eye tracker, how reliably they maintained fixation is unknown, so this remains an issue. Furthermore in terms of general visual input, the use of virtual acoustics in isolation carries with it an inevitable mismatch between audition and vision. The impact of such a mismatch was mitigated somewhat in our experiment because the listeners were seated at the center of a ring of 24 loudspeakers, meaning that there was always a loudspeaker within 15° of the simulated acoustic angle. That said, future work will have to examine the important role of vision and eye movements in this phenomenon.

Another potential factor is that of proprioception: when the head turns, the flexing of muscles and the changing angle of the neck produces somatosensory stimulation that may also be integrated into both the percept of motion and of sound source location. Indeed it has been shown that straining the head against the rotation of a chair can abolish the audiogyral illusion (Lester and Morant, 1970). On the other hand, it has been demonstrated that proprioception plays a lesser role than that of the vestibular system in the discrimination of front/back location (Kim et al., 2013). Regardless of whether such input may be integrated into spatial auditory perception, since we were not able to replicate the natural movements of our listeners using a programmable motion-controlled chair, proprioception remains out of the scope of the current study. It should be noted that the movements in our study consisted of roughly sinusoidal back and forth rotations, necessarily involving angular acceleration. It is unknown whether the effects observed in our study would be the same for a listener turning at a constant rotational velocity and thereby reducing both proprioceptive and vestibular cues, so this too remains an open question. However, despite the fact that our study could not take into account eye movements, constant rotation, or proprioceptive input, we argue that our results are nevertheless attributable to a basic difference in the processing of self motion and world motion.

## Conclusions

We found that for all age groups and levels of hearing impairment, the MMAA during self motion was smaller than during source motion. Thus listeners are more accurate at processing self-generated acoustic motion than source generated-motion. These results suggest that auditory spatial perception is at the very least continually informed by self motion; that is, listeners are engaged in constant and ongoing comparison between their own movement and the apparent movement of the auditory world. Furthermore, we find that the data are consistent with the hypothesis that self motion is at least partially compensated for, providing a more stable backdrop against which spatial location and “real” movement may be better discriminated.

### Conflict of interest statement

The authors declare that the research was conducted in the absence of any commercial or financial relationships that could be construed as a potential conflict of interest.

## Footnotes

^1^It should be noted that linear interpolation of BRIRs can never result in a BRIR that is identical to one actually measured at the interpolated location, but the fine-grained nature of the database we used minimized this problem.

^2^Another factor that could contribute to an underestimation of the actual self-motion advantage is that of an order effect. By necessity of the study design the self-motion condition had to be run before the world-motion condition in each block. If listeners performed better over time, then the source-motion condition could be easier on average for the subject.
